# Gap-free telomere-to-telomere genome assembly of stone flounder (*Kareius bicoloratus*)

**DOI:** 10.1038/s41597-026-07661-x

**Published:** 2026-06-22

**Authors:** Weiwei Zheng, Kaiqiang Liu, Tao Yang, Dong Xu, Zhihong Liu, Wei Yuan, Yan Liu, Tengteng Wang, Huizong Han, Xueyan Fang, Wenteng Xu, Songlin Chen

**Affiliations:** 1 State Key Laboratory of Mariculture Biobreeding and Sustainable Goods, Yellow Sea Fisheries Research Institute, Chinese Academy of Fishery Sciences, Qingdao, Shandong 266071 China; 2 Laboratory for Marine Fisheries Science and Food Production Processes, Qingdao Marine Science and Technology Center, Qingdao, Shandong 266237 China; 3 Shandong Marine Resource and Environment Research Institute, Yantai, Shandong 264006 China; 4 Bureau of Agriculture and Rural Affairs of Laoshan District, Qingdao City, Qingdao, Shandong 266100 China

## Abstract

Stone flounder (*Kareius bicoloratus*) is an emerging commercially important flatfish species in China, Japan, Korea, and Taiwan, because of its significant industrial value. However, no stone flounder genome sequence was reported to date, which greatly hinders the research on genetics and evolutionary genomics. In this study, we successfully assembled the first gap-free telomere-to-telomere (T2T) genome of stone flounder combining Illumina short-reads sequencing, PacBio HiFi sequencing, Hi-C sequencing, and ONT ultra-long sequencing. The genome spans 587.80 Mb with a contig N50 of 25.64 Mb. 98.58% of the genome sequences were assembled onto 24 chromosomes without gaps. Telomeres were identified at both ends of 8 chromosomes and at one end of 15 chromosomes. We identified 128.10 Mb repetitive sequences and 22,312 protein-coding genes, among which 98.03% of the coding genes were functionally annotated. BUSCO assessment, mapping ratio, and quality value (QV) evaluation collectively indicated the high quality and completeness of the genome assembly. This work advances the progress of evolutionary genomics, genetics and molecular genetic breeding research on stone flounder.

## Background & Summary

Stone flounder (*Kareius bicoloratus*, FishBase ID: 8557), a member of the family Pleuronectidae of the order Pleuronectiformes, is naturally distributed in the East China Sea and the coastal waters of southern Japan^1,2^. In recent years, it has become a commercially important species in China, Japan, Korea, and Taiwan, because of its industrial values, such as low temperature tolerance^3^, adaptation to euryhaline conditions^4^, high market demand^2^, and excellent meat quality and flavor^5^.

At present, a large number of studies on stone flounder mainly focus on early life history^6^, biology^7^, feeding habits^8–11^, nutritional composition^12,13^, growth^4,11^, salinity tolerance^14,15^, population structure^16^, sperm cryopreservation^2^, hybridization^3,17^, and molecular marker^18^. However, to date, no high-quality reference genome of stone flounder has been reported, which has greatly hindered the comprehensive research in genetics, evolutionary genomics and functional genomics. Thus, there is an urgent need to assemble a high-quality genome of stone flounder.

The rapid development of sequencing technologies and assembly algorithms, especially long-read sequencing technologies like PacBio (Single Molecule Real Time) SMRT sequencing, have made it possible to obtain high-quality reference genome. It is worth mentioning that several high-quality reference genomes of flatfish have been reported, including *Verasper variegatus*^19^, Japanese flounder (*Paralichthys olivaceus*)^20^, turbot (*Scophthalmus maximus*)^21^, and starry flounder (*Platichthys stellatus*)^22^. Furthermore, recently, the application of Oxford Nanopore Technologies (ONT) ultra-long sequencing has successfully assisted in assembling multiple T2T reference genomes of fish, such as marbled flounder (*Pseudopleuronectes yokohamae*)^23^, Chinese sea bass (*Lateolabrax maculatus*)^24^, the giant grouper (*Epinephelus lanceolatus*)^25^, the tomato hind (*Cephalopholis sonnerati*)^26^, Asian icefish (Protosalanx chinensis)^27^, humphead wrasse (*Cheilinus undulatus*)^28^, Amur minnow (*Phoxinus lagowskii*)^29^, rock carp (*Procypris rabaudi*)^30^, African catfish (*Clarias gariepinus*)^31^, Yadong trout (*Salmo trutta*)^32^, and zig-zag eel (*Mastacembelus armatus*)^33^, blackspot tuskfish (*Choerodon schoenleinii*)^34^, blackhead seabream (*Acanthopagrus schlegelii*)^35^, glass catfish (*Kryptopterus vitreolus*)^36^ and X-ray tetra (*Pristella maxillaris*)^37^.

**Fig. 1:**
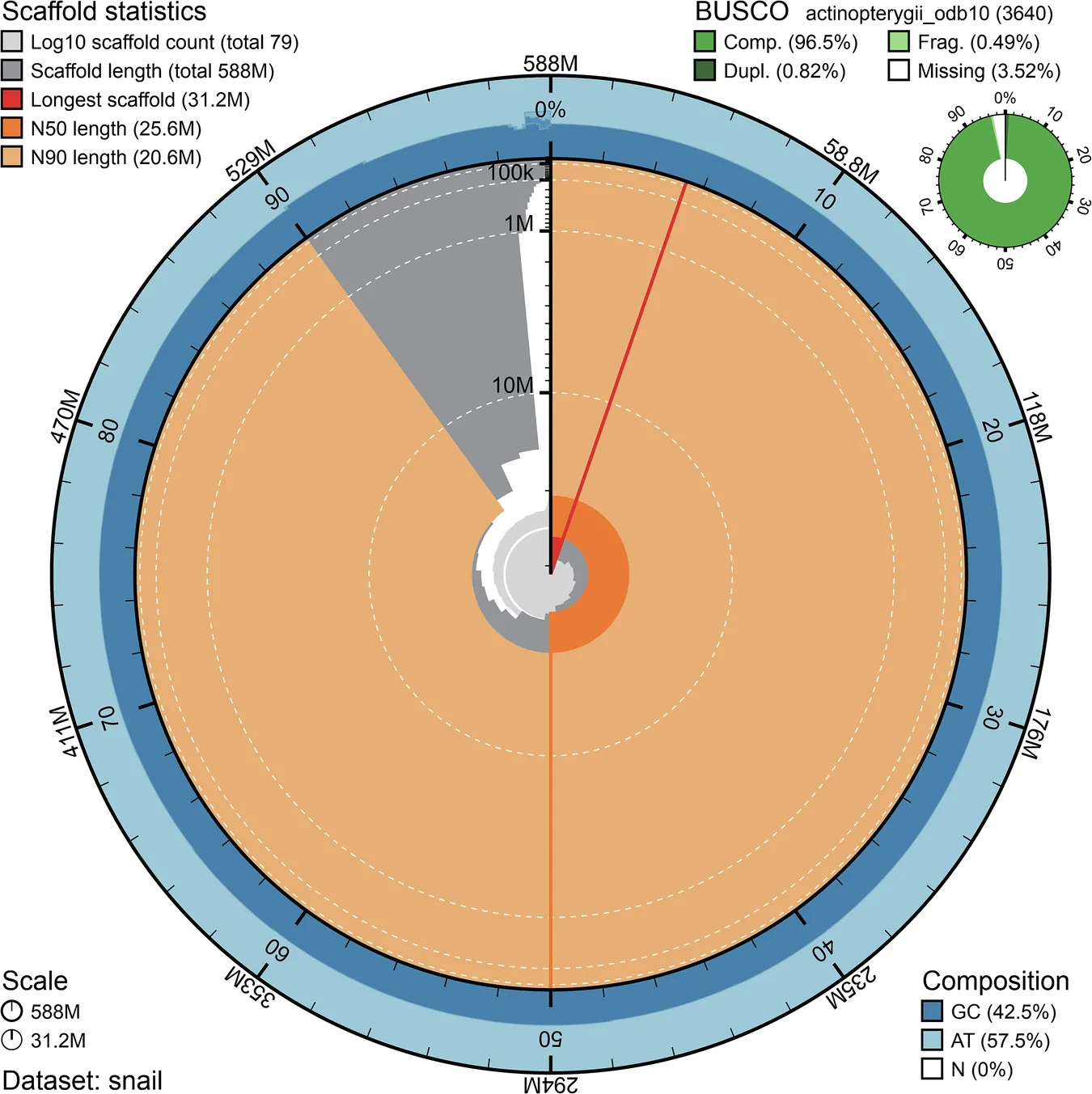
The genome snail plot of *K. bicoloratus*.

In this study, we reported a gap-free T2T genome of stone flounder combing Illumina short-read sequencing, PacBio high-fidelity (HiFi) sequencing, and high-throughput chromosome conformation capture (Hi-C) sequencing, and ONT ultralong read sequencing technologies (Fig. 1). To our knowledge, this is the first reported genome sequence of stone flounder so far. This high-quality genome assembly will not only advance the evolutionary genomics and genetic research of flatfish, but also provide a robust foundation for the development of molecular genetic breeding in stone flounder.

## Methods

### Sample collection and genome sequencing

For genome sequencing, a two-year-old artificially farmed female stone flounder weighing 452.08 g with a total length of 28.36 cm was sampled from Laizhou Mingbo Aquatic Co., Ltd., Yantai, Shandong, China. Muscle tissues were collected for Genomic DNA (gDNA) extraction. Eight different tissues such as gill, gonad, heart, kidney, liver, muscle, skin, and spleen were sampled for RNA extraction. All samples collected in this study were approved by the the Animal Care and Use Committee of Yellow Sea Fisheries Research Institute, Chinese Academy of Fishery Sciences (CAFS). gDNA was extracted using the TIANamp Marine Animals DNA Kit (Tiangen, China) for Illumina short-read sequencing and Hi-C sequencing. For PacBio HiFi sequencing and ONT ultra-long sequencing, gDNA was extracted using the standard sodium dodecyl sulfate (SDS) extraction method. For RNA-seq used for coding gene annotation, RNA was extracted using the RNA Easy Fast Tissue/Cell Kit (Tiangen, China). All gDNA was quantified and qualified using agarose gel electrophoresis, NanoDrop One Spectrophotometers (Thermo Fisher Scientific, USA), and Qubit 3.0 Fluorometer (Life Technologies, USA) to meet the requirements of library construction. RNA was quantified and qualified by agarose gel electrophoresis, NanoDrop One Spectrophotometers, Qubit 3.0 Fluorometer, and Agilent 2100 (Agilent Technologies, Germany).

For Illumina short-read sequencing, a 200–400 bp short-read library was constructed using Illumina DNA PCR-Free Prep kit (Illumina, USA) following the standard protocol, and the qualified library was sequenced on Illumina Novaseq 6000 platform with 150 bp pair-end (PE) mode. As a result, a total of 44.15 Gb (75.11×) short-read sequencing data was generated (Table 1).

**Table 1:** Summary of sequencing data for *K. bicoloratus* genome assembly.

Library Type	Sequencing Platform	Tissue	Raw data (Gb)	Depth (×)	Average Read Length (bp)
Illumina	Illumina Novaseq 6000	Muscle	44.15	75.11	150
Pacbio HiFi	PacBio Sequel II	Muscle	18.38	31.27	17,343
Hi-C	Illumina Novaseq 6000	Muscle	177.54	302.04	150
ONT	Oxford Nanopore PromethION	Muscle	23.41	39.83	36,556
RNA-seq	Illumina Novaseq 6000	spleen	7.86	—	150
RNA-seq	Illumina Novaseq 6000	gill	7.48	—	150
RNA-seq	Illumina Novaseq 6000	gonad	6.69	—	150
RNA-seq	Illumina Novaseq 6000	liver	7.39	—	150
RNA-seq	Illumina Novaseq 6000	muscle	8.20	—	150
RNA-seq	Illumina Novaseq 6000	skin	7.48	—	150
RNA-seq	Illumina Novaseq 6000	heart	7.40	—	150
RNA-seq	Illumina Novaseq 6000	kidney	6.91	—	150
Iso-seq	PacBio Sequel IIe	Mixed tissue	93.43	—	1,734

For PacBio HiFi sequencing, qualified genomic DNA was used to construct a SMRTbell library using the SMRTbell Prep Kit 3.0 (Pacbio Biosciences, CA, USA) according to the manufacturing protocols, and the qualified library was then performed on PacBio Sequel II platform for sequencing with the Circular Consensus Sequencing (CCS) mode. Finally, 18.38 Gb (31.27×) PacBio HiFi sequencing data were obtained, with an average read length of 17.34 kb and N50 read length of 17.29 kb (Table 1).

The Hi-C library construction process includes formaldehyde crosslinking, cell lysis, enzymatic digestion, end repair and biotin labeling, blunt-end ligation, crosslinking reversal, and DNA purification^38^. Then, the qualified Hi-C library was sequenced on Illumina Novaseq 6000 platform with 150 bp PE mode. As a result, 177.54 Gb (302.04×) Hi-C sequencing data was generated (Table 1).

For ONT ultra-long sequencing, the library was prepared using the Oxford Nanopore SQK-ULK001 ligation kit, and the purified library was loaded onto primed flow cell and sequenced on PromethION sequencer (Oxford Nanopore Technologies, Oxford, UK). Finally, 23.41 Gb (39.83×) of ONT ultra-long sequencing data were obtained (Table 1). The average read length and read N50 length are 36.56 kb and 52.72 kb, respectively.

For assist in coding genes annotation, RNA from eight different tissues, including spleen, gill, gonad, liver, muscle, skin, heart, and kidney, was extracted. Eight pair-ended sequencing library was constructed and sequenced on Illumina Novaseq 6000 platform. As a result, a total of 59.41 Gb RNA-seq data were generated (Table 1). In addition, RNA isolated from the above eight different tissues was equally mixed into a total RNA for Isoform-sequencing (Iso-seq). Qualified RNA was used for library construction with SMRTbell prep kit 3.0. Then, the library was sequenced on the PacBio Sequel IIe platform, resulting in 93.43 Gb of Iso-seq data (Table 1).

### Genome assembly and telomere identification

**Fig. 2:**
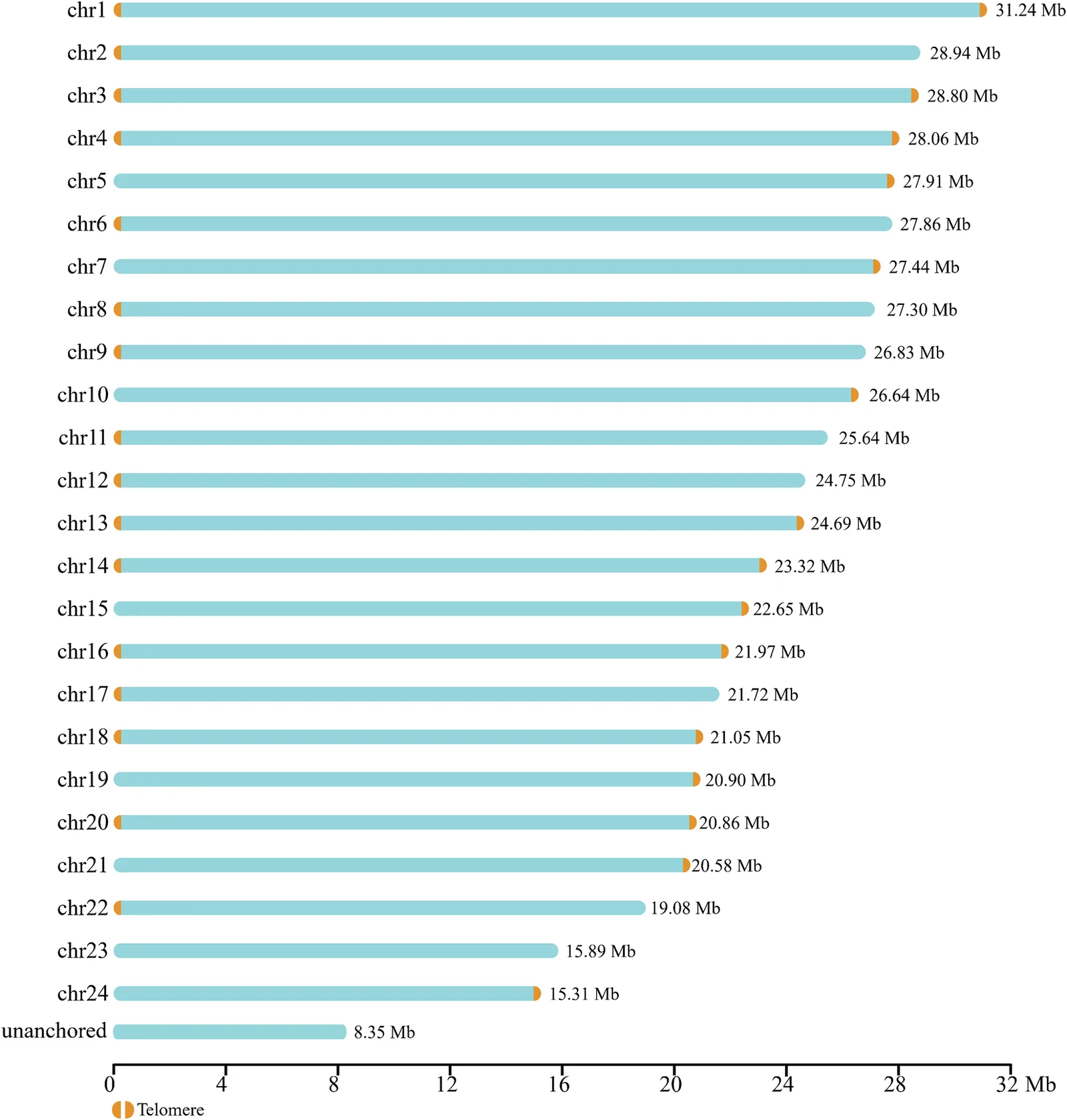
Distribution map of telomeres on the T2T genome of *K. bicoloratus*.

The primary contigs were initially assembled using Hifiasm^39^ (v0.19.9) with the PacBio HiFi, Hi-C, and ONT ultralong sequencing data described above. Subsequently, the draft genome was anchor to 24 chromosomes using Yahs pipeline^40^ (v.1.2a.1). Then, ONT data was used to fill gaps with TGS-GapCloser^41^ (v1.2.1) to lengthen the contigs. Thereafter, Pilon^42^ (v1.23) was employed to polish the genome assembly with Illumina short-read data. Finally, a gap-free genome assembly of stone flounder with a length of 587.80 Mb was generated, and its contig N50 was 25.64 Mb (Fig. 1, Table 2). We further identified the telomeric repeat motifs (CCCTAA/TTAGGG) at both ends of each chromosome in the stone flounder genome sequence using quarTeT^43^ (v1.2.5). As a result, a total of 31 telomeres were identified, and telomeres were detected on both ends of 8 chromosomes (Fig. 2, Table 3).

**Table 2:** Statistics of genome assembly in *K. bicoloratus*.

	GCA_054131325.1
Total genome length (Mb)	587.80
Total chromosome length (Mb)	579.44
Number of chromosome	24
Number of Scaffolds	79
Contig N50 (Mb)	25.64
Scaffold N50 (Mb)	25.64

**Table 3:** Statistics of chromosomes and telomeres in *K. bicoloratus*.

Chromosome	Length(bp)	Number of Telomeres	Number of gaps
chr1	31,241,952	2	0
chr2	28,942,177	1	0
chr3	28,797,775	2	0
chr4	28,063,851	2	0
chr5	27,908,431	1	0
chr6	27,864,981	1	0
chr7	27,438,496	1	0
chr8	27,302,822	1	0
chr9	26,826,532	1	0
chr10	26,643,321	1	0
chr11	25,639,765	1	0
chr12	24,751,850	1	0
chr13	24,692,628	2	0
chr14	23,323,095	2	0
chr15	22,651,375	1	0
chr16	21,969,867	2	0
chr17	21,720,195	1	0
chr18	21,054,073	2	0
chr19	20,897,406	1	0
chr20	20,859,748	2	0
chr21	20,584,739	1	0
chr22	19,075,680	1	0
chr23	15,887,518	0	0
chr24	15,305,360	1	0
Unplaced	8,353,207	—	—

### Repeat annotation

A combined strategy involving homologous prediction and *de novo* prediction were employed to identify the whole genome repeats. The homology prediction was conducted using RepeatMasker software^44^ (v4.1.2) and its in-house scripts (RepeatProteinMask) (v4.1.2) with default parameters based on Repbase database^45^ (http://www.girinst.org/repbase) (v21.01) to extracted repeat regions. *de novo* repetitive elements database was built by RepeatModeler^46^ (v2.0.3) with default parameters. Subsequently, integrating the Repbase database with the *de novo* repetitive elements database library to generate a non-redundant library through uclust processing. Then, the custom library was supplied to RepeatMasker for DNA-level repeat identification. Ultimately, a total of 128.10 Mb of non-redundant repetitive sequences were predicted, constituting approximately 21.79% of the stone flounder genome (Table 4). Among these repeats, short interspersed nuclear elements (SINEs), long interspersed elements (LINEs), long terminal repeats (LTRs), and DNA transposons accounted for 0.26%, 3.05%, 2.14%, and 7.82% of the genome, respectively (Table 4).

**Table 4:** Classification statistics of repeated elements in *K. bicoloratus*.

Type	Length(bp)	Percentage(%)
SINE	1,507,115	0.26
LINE	17,647,701	3.05
LTR	12,392,485	2.14
DNA	45,326,398	7.82
Unknown	51,228,823	8.52
Total	128,102,522	21.79

### Protein-coding gene prediction and functional annotation

**Fig. 3:**
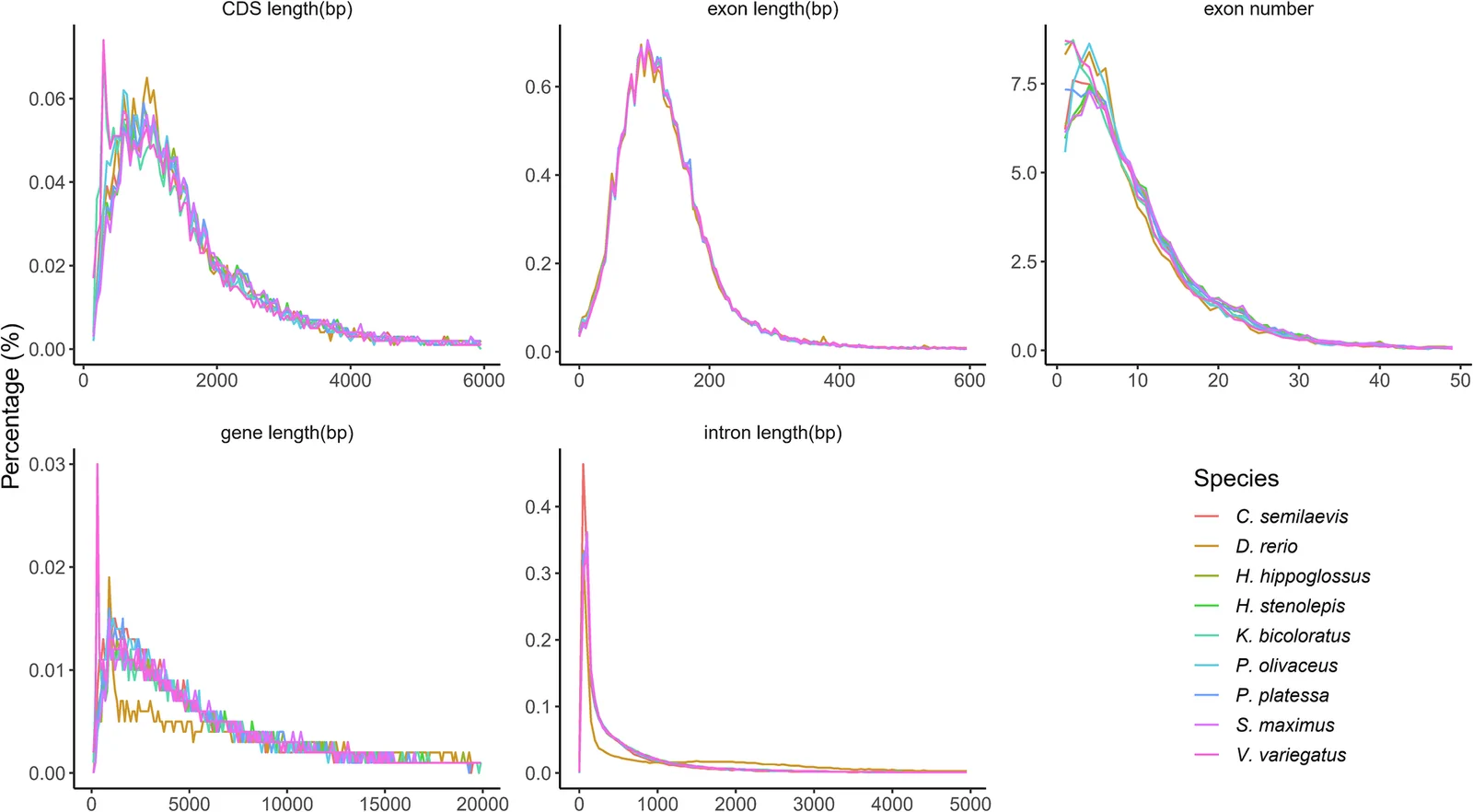
Distribution of the gene length, coding sequence (CDS) length, exon length, and intron length among *K. bicoloratus*, *Verasper variegatus*, *Pleuronectes platessa*, *Hippoglossus hippoglossus*, *H. stenolepis*, *Scophthalmus maximus*, *Paralichthys olivaceus*, *Cynoglossus semilaevis*, and *Danio rerio*.

*De novo* prediction, homology-based prediction, transcriptomic-assisted prediction, and evidence-based prediction approaches were employed to protein-coding gene annotation. For *de novo* prediction, Augustus^47^ (v3.5) and SNAP^48^ (v2013.11.29) with default parameters were used for the gene structure prediction. For homology-based prediction, homologous protein sequences of *Verasper variegatus*, *Pleuronectes platessa*, *Hippoglossus hippoglossus*, *H. stenolepis*, *Scophthalmus maximus*, *Paralichthys olivaceus*, *Cynoglossus semilaevis*, and *Danio rerio* were downloaded from NCBI or Ensembl. Protein sequences were aligned to the stone flounder genome using TblastN (v2.2.26; E-value ≤ 1e–5), and then the matching proteins were aligned to the homologous genome sequences for accurate spliced alignments with GeneWise^49^ (v2.4.1) to predict gene structure contained in each protein region. For transcriptomic-assisted prediction, RNA-seq reads from Illumina and Pacbio Sequel IIe sequencing were aligned to genome using Hisat^50^ (v2.2.1) with default parameters to identify exons region and splice positions. The alignment results were then used as input for genome-based transcript assembly by Stringtie^51^ (v2.2.1) with default parameters and validated by PASA (v2.5.2) (https://github.com/PASApipeline/PASApipeline). Additionally, to ensure high-quality structural annotation of the genome, we also employed an evidence-based approach using EGAPx^52^ (prediction of gene structures for eukaryotic genomes, v0.5.0; https://github.com/ncbi/egapx). RNA-seq data generated in this study were utilized as transcriptomic evidence to support gene modeling. The EGAPx pipeline integrated short reads alignments and assembled transcripts to define gene structures, including exon-intron boundaries and coding sequences (CDS). The EvidenceModeler (EVM)^53^ pipeline (v1.1.1) was employed to integrate all the protein-coding genes predicted by the above four methods and the result was corrected using PASA. Finally, a non-redundant reference gene set including 22,312 coding genes was obtained with an average gene length of 12,246.75 bp (Table 5). The distribution patterns of gene length, coding sequence (CDS) length, exon length, and intron length in stone flounder and eight other teleost fish species were similar (Fig. 3).

**Table 5:** Statistics of predicted protein-coding genes in *K. bicoloratus*.

Gene set		Gene number	Average gene length (bp)	Average CDS length (bp)	Average exon per gene	Average exon length (bp)	Average intron length (bp)
*Denovo*	SNAP	53,752	13,626.05	970.91	6.59	147.25	2,262.40
	AUGUSTUS	30,736	7,044.60	1,309.56	7.41	176.79	895.04
Homolog	*Hippoglossus stenolepis*	20,251	11,886.67	1,806.37	10.43	173.19	1,068.94
	*Paralichthys olivaceus*	21,288	10,252.09	1,634.29	9.38	174.18	1,028.03
	*Cynoglossus semilaevis*	18,867	11,040.62	1,731.22	9.78	176.98	1,060.05
	*Danio rerio*	18,775	10,212.36	1,612.01	9.04	178.36	1,070.00
	*Pleuronectes platessa*	20,186	11,491.85	1,782.16	10.31	172.84	1,042.80
	*Scophthalmus maximus*	19,932	11,717.60	1,818.85	10.29	176.67	1,064.97
	*Hippoglossus hippoglossus*	20,203	11,882.42	1,802.26	10.43	172.75	1,068.62
	*Verasper variegatus*	21,982	10,063.52	1,574.80	9.10	172.98	1,047.46
RNAseq	transcripts	40,014	14,671.98	3,107.86	10.91	284.87	1,166.94
	PASA	35,293	7,788.07	1,246.99	7.78	160.21	964.25
EGAPx	EGAPx	21,502	14,627.54	1,859.85	10.77	172.73	1,180.94
EVM	EVM	29,227	8,747.14	1,369.12	7.94	172.47	1,063.36
Pasa-update	pasaupdate	28,805	9,022.04	1,403.32	8.11	173.08	1,071.88
Final set	Final	22,312	12,246.75	1,651.00	9.98	239.97	1,097.48

The coding genes predicted above were annotated by aligning them to databases, such as InterPro^54^, GO^55^, KEGG^56^, Swissprot^57^, Pfam^58^, and NR^59^, using DIAMOND^60^ (v2.1.8) (E-value = 1e–5), InterProScan^61^ (v5.75–106.0), or the corresponding built-in software. In total, 21,872 genes were annotated for at least one homologous hit by searching against these public databases, accounting for 98.03% of all inferred genes (Table 6).

**Table 6:** Statistics of functional annotation of protein-coding genes in *K. bicoloratus*.

Type		Number	Percent (%)
Total		22,312	
Annotated	NR	21,350	95.69
	Swissport	19,992	89.60
	KEGG	19,154	85.85
	InterPro	21,454	96.15
	Pfam	18,519	83.00
	GO	15,033	67.38
Unannotated		440	1.97

For non-coding RNAs annotation, 5,314 tRNAs were predicted using the program tRNAscan-SE^62^ (v2.0.12). 14,041 rRNAs highly conserved were identified by blast (v2.2.26). Furthermore, 2,510 miRNAs and 2,117 snRNAs were identified by searching against the Rfam database using Infernal^63^ (v1.1.4) (Table 7).

**Table 7:** Statistics of non-coding RNA in *K. bicoloratus*.

Type		Copy	Average length (bp)	Total length (bp)	% of genome
miRNA		2,510	108.787	273,056	0.047
tRNA		5,314	75.433	400,851	0.069
rRNA	rRNA	14,041	113.690	1,596,317	0.275
	18S	130	269.300	35,009	0.006
	28S	280	218.018	61,045	0.011
	5.8S	12	139.667	1,676	0.000
	5S	13,619	110.036	1,498,587	0.259
snRNA	snRNA	2,117	149.401	316,281	0.055
	CD-box	187	127.797	23,898	0.004
	HACA-box	96	174.812	16,782	0.003
	splicing	1,786	149.449	266,916	0.046
	scaRNA	47	183.596	8,629	0.001

## Data Records

The Illumina sequencing data, PacBio HiFi sequencing data, Hi-C sequencing data, and ONT ultra-long sequencing data have been uploaded to NCBI Sequence Read Archive (SRA) database with accession number SRP636034^64^. The RNA-seq and Iso-seq data used for coding genes annotation have also been uploaded to NCBI Sequence Read Archive (SRA) database with accession number SRP638591^65^. The genome assembly has been submitted to NCBI under accession number GCA_054131325.1^66^. The genome annotation file, gene CDS, and protein data have been deposited at Figshare^67^.

## Technical Validation

### Completeness and quality assessment of genome assembly

The completeness of the stone flounder genome assembly was evaluated using BUSCO (v5.3.2)^68^ with the actinopterygii_odb10 database including 3,640 BUSCOs. Of these, 3,512 (96.5%) complete BUSCOs including 3,482 (95.7%) single-copy BUSCOs and 30 (0.8%) duplicated BUSCOs were identified. Only 18 (0.5%) fragmented BUSCOs and 110 (3.0%) missing BUSCOs were detected. The genome quality value (QV) was accessed by Merqury^69^, and the QV score was 48.41, highlighting a high-quality assembly. Furthermore, the accuracy rate was evaluated by mapping the sequencing data to the genome assembly using bwa^70^ (v0.7.17) and minimap2^71^ (v2.17). The mapping rates were 95.63% and 99.24% for Illumina sequencing data and PacBio HiFi sequencing data, respectively.

### Evaluation of the gene annotation

The accuracy of gene annotation was evaluated using BUSCO (v5.3.2) on the basis of actinopterygii_odb10 database containing 3,640 BUSCOs. The results showed that 3,432 (94.3%) complete BUSCOs, containing 3,403 (93.5%) single-copy and 29 (0.8%) duplicated BUSCOs, were detected, 22 (0.6%) fragmented BUSCOs and 186 (5.1%) missing BUSCOs were identified.

## Data Availability

The Illumina sequencing data, PacBio HiFi sequencing data, Hi-C sequencing data, and ONT ultra-long sequencing data are available at NCBI SRA database under accession number SRP636034 (https://identifiers.org/ncbi/insdc.sra:SRP636034). The RNA-seq and Iso-seq data used for coding genes annotation are available at NCBI SRA database with accession number SRP638591 (https://identifiers.org/ncbi/insdc.sra:SRP638591). The genome assembly is available at NCBI GenBank with accession number GCA_054131325.1 (https://identifiers.org/ncbi/insdc.gca:GCA_054131325.1). The genome annotation file, gene CDS, and protein data are available at the Figshare repository (https://doi.org/10.6084/m9.figshare.31175527.v1).

## Code availability

All software and tools were used in this study in accordance with the instructions and protocols provided by the respective software developers. The software versions and corresponding parameters applied have been described in the Methods section, and default parameters were used if no parameter was described. No custom code was used in this work.
